# Individual and Neighborhood Socioeconomic Status and the Association between Air Pollution and Cardiovascular Disease

**DOI:** 10.1289/EHP199

**Published:** 2016-05-03

**Authors:** Gloria C. Chi, Anjum Hajat, Chloe E. Bird, Mark R. Cullen, Beth Ann Griffin, Kristin A. Miller, Regina A. Shih, Marcia L. Stefanick, Sverre Vedal, Eric A. Whitsel, Joel D. Kaufman

**Affiliations:** 1 Department of Epidemiology, School of Public Health, University of Washington, Seattle, Washington, USA; 2 Department of Environmental and Occupational Health Sciences, School of Public Health, University of Washington, Seattle, Washington, USA; 3 RAND Corporation, Santa Monica, California, USA; 4 Department of Internal Medicine, Stanford University School of Medicine, Stanford, California, USA; 5 RAND Corporation, Arlington, Virginia, USA; 6 Department of Medicine, Stanford Prevention Research Center, Stanford University School of Medicine, Stanford, California, USA; 7 Department of Epidemiology, UNC Gillings School of Global Public Health, Chapel Hill, North Carolina, USA; 8 Department of Medicine, University of North Carolina at Chapel Hill, Chapel Hill, North Carolina, USA

## Abstract

**Background:**

Long-term fine particulate matter (PM2.5) exposure is linked with cardiovascular disease, and disadvantaged status may increase susceptibility to air pollution-related health effects. In addition, there are concerns that this association may be partially explained by confounding by socioeconomic status (SES).

**Objectives:**

We examined the roles that individual- and neighborhood-level SES (NSES) play in the association between PM2.5 exposure and cardiovascular disease.

**Methods:**

The study population comprised 51,754 postmenopausal women from the Women’s Health Initiative Observational Study. PM2.5 concentrations were predicted at participant residences using fine-scale regionalized universal kriging models. We assessed individual-level SES and NSES (Census-tract level) across several SES domains including education, occupation, and income/wealth, as well as through an NSES score, which captures several important dimensions of SES. Cox proportional-hazards regression adjusted for SES factors and other covariates to determine the risk of a first cardiovascular event.

**Results:**

A 5 μg/m3 higher exposure to PM2.5 was associated with a 13% increased risk of cardiovascular event [hazard ratio (HR) 1.13; 95% confidence interval (CI): 1.02, 1.26]. Adjustment for SES factors did not meaningfully affect the risk estimate. Higher risk estimates were observed among participants living in low-SES neighborhoods. The most and least disadvantaged quartiles of the NSES score had HRs of 1.39 (95% CI: 1.21, 1.61) and 0.90 (95% CI: 0.72, 1.07), respectively.

**Conclusions:**

Women with lower NSES may be more susceptible to air pollution-related health effects. The association between air pollution and cardiovascular disease was not explained by confounding from individual-level SES or NSES.

**Citation:**

Chi GC, Hajat A, Bird CE, Cullen MR, Griffin BA, Miller KA, Shih RA, Stefanick ML, Vedal S, Whitsel EA, Kaufman JD. 2016. Individual and neighborhood socioeconomic status and the association between air pollution and cardiovascular disease. Environ Health Perspect 124:1840–1847; http://dx.doi.org/10.1289/EHP199

## Introduction

Large population studies have routinely demonstrated that exposure to air pollution is associated with increased risk of cardiovascular morbidity and mortality (Brook et al. 2010). Low-socioeconomic status (SES) has also consistently been identified as a risk factor for cardiovascular disease (CVD) (Elo 2009). In addition, SES putatively co-varies with the spatial distribution of air pollution (Hajat et al. 2015). In this study, we set out to address two different questions regarding the role of SES in the air pollution–CVD relationship.

Our first aim was to answer a substantive question of whether individuals with low SES are more susceptible to the effects of air pollution on CVD. This question is crucial in informing air quality standards sufficient to protect the health of sensitive groups. We addressed this question by testing whether individual or neighborhood SES are effect modifiers of the air pollution–CVD relationship. Extant literature provides mixed evidence of effect modification by SES on the association between air pollution and health outcomes, including CVD. Low-SES individuals may be more susceptible to adverse effects of air pollution because they have poorer health resulting from reduced material resources, have higher psychosocial stress, and exhibit more individuals risk factors such as unhealthy behaviors and lifestyles (Elo 2009; O’Neill et al. 2003).

Our second question addresses the important methodological problem of whether SES confounds the association between air pollution and CVD. Confounding by SES is particularly concerning because low SES is a strong risk factor for CVD (Elo 2009) and also co-varies spatially with air pollution. Some North American studies have reported that communities with low SES are more likely to be exposed to higher concentrations of air pollution (Hajat et al. 2015), whereas, European research has been mixed (Hajat et al. 2015). The direction of confounding may depend on how SES co-varies with air pollution in the study population.

Epidemiological studies of air pollution health effects commonly include some measures of SES, such as individual-level education or income, but few incorporate multiple levels of SES. Both individual-level SES and neighborhood-level SES (NSES) are independently related to PM_2.5_ (Chaix et al. 2006; Hajat et al. 2013). NSES may have greater impact on estimated associations of air pollution on mortality than does individual-level SES (Næss et al. 2007). Not controlling for both levels of SES may lead to potential residual confounding. In addition, individual-level and contextual NSES may increase susceptibility to air pollution-related health outcomes via different processes. For instance, individual poor health status (e.g., diabetes and obesity) may act in synergy with air pollution exposure to promote CVD (O’Neill et al. 2003). On the neighborhood level, contextual factors such as lower-housing stock may increase co-exposure of other pollutants to induce greater susceptibility to harmful effects of air pollution (O’Neill et al. 2003).

SES has been characterized as a multidimensional concept often operationalized by measuring three specific domains: education, occupation, and income/wealth—each having different effects at various times in the life course (Elo 2009). For example, some have proposed that education affects health by promoting accumulation of knowledge regarding health-promoting behaviors and technologies and by improving decision-making and problem-solving skills (Elo 2009). As for occupational class, those working in higher occupational class positions tend to have less exposure to potentially harmful chemicals and pollutants present in the workplace (O’Neill et al. 2003). Lastly, income and wealth are financial resources that enable access to health-generating resources (such as good quality housing in safe neighborhoods) and access to higher quality health care (Elo 2009). The three domains are also interrelated because educational attainment influences subsequent occupation and income. Thus, epidemiological studies involving SES should include measures that reflect various domains of SES.

Unlike many data sets, the Women’s Health Initiative (WHI) Observational Study has available a unique set of SES indicators on both the individual and neighborhood levels that span the three domains of SES: education, occupation, and income/wealth. This is a substantial methodological improvement in measuring SES. In this article, we examine the roles that individual-level SES and NSES play in the association between fine particulate matter (PM_2.5_) and incident cardiovascular events in the longitudinal WHI Observational Study.

## Methods

### Study Population

The WHI Observational Study enrolled 93,676 postmenopausal women 50–79 years old from 40 centers throughout the United States between 1993 and 1998. Eligible women included those who provided written informed consent, who planned to stay in the area, and who were free of conditions that might interfere with follow-up. The study design and participant characteristics were described previously (Women’s Health Initiative Study Group 1998; Hays et al. 2003; Langer et al. 2003). In the observational study, 93,676 women were screened at baseline to obtain information on demographics, lifestyle, medical history, cardiovascular risk factors, and anthropometric and blood pressure measurements (Langer et al. 2003). Annual mailed questionnaires collected updates on health outcomes. In this analysis, participants were followed from baseline until the end of follow-up of the main cohort in September 2005 (mean follow-up 7.6 years). Participants of this study were postmenopausal women > 50 years old at baseline who were better educated than women of the same age in the U.S. general population, limiting our ability to generalize our results to the overall population.

The current analysis was restricted to participants free of CVD (myocardial infarction, congestive heart failure, coronary revascularization, and stroke) at baseline with at least one PM_2.5_ prediction resolved to the street (i.e., higher resolution than ZIP code centroid) over the study period. Of the 93,676 participants, 18,576 had CVD and 2,006 had missing CVD status at baseline and were excluded. A further 17,115 participants were excluded due to missing covariates. Of the remaining 55,979 women, 1,493 had completely missing PM_2.5_ predictions and 2,732 had geocodes not resolved to the street. These categories were not mutually exclusive, and our final analytic sample included 51,754 women. Those excluded had lower SES and were more likely to be nonwhite, smokers, and diabetics. However, the analytic sample is generally representative of the baseline sample of women who were free of CVD, except for having more white participants than the baseline sample (86.3% vs. 83.6%). All standardized mean differences comparing important measures (including exposure and CVD incidence) of complete cases to the original sample of eligible participants were < 0.1 (see Table S1). Thus, all important measures were well-balanced between the complete cases and original sample. The proportion of missing data ranged from 0.05% to 10.6%.

### PM_2.5_ Exposures

All known participant home addresses over the follow-up period were geocoded. For each address, the point-specific annual average PM_2.5_ concentration was predicted using U.S. Environmental Protection Agency’s (EPA) Air Quality System (AQS) and Interagency Monitoring of Protected Visual Environments (IMPROVE) monitoring data for the year 2000 and used to represent ambient PM_2.5_ concentrations at that address over the entire follow-up. The year 2000 was selected because it represented an early year of complete national PM_2.5_ monitoring and a representative year of the follow-up period. Relative concentrations of particulate pollution were largely consistent for study locations throughout the study period (Miller et al. 2007). In addition, analysis from the American Cancer Society’s study demonstrated that PM_2.5_ was strongly correlated between sites during a 20-year period and the hazard function was not time-dependent—suggesting that fine particulate matter measured at any point over the study period is a reasonable surrogate for long-term particulate matter exposure (Abrahamowicz et al. 2003). Likelihood-based ambient point-specific PM_2.5_ predictions at participant residences were obtained using a regionalized national universal kriging model that included over 200 geographic covariates reduced via partial least squares techniques (Sampson et al. 2013). This approach resulted in a high level of cross-validated accuracy of prediction with an overall *R* ^2^ of 0.88.

To calculate time-varying PM_2.5_ exposure, data were split on each time that a first cardiovascular event occurred, generating multiple records for each address for each participant. For each record, PM_2.5_ exposure was calculated as an average of the current and all previous PM_2.5_ predictions weighted by time spent at each residence. Splitting the data allowed us to calculate exposures that incorporated information before a cardiovascular event or censorship. The exposure was only time-varying in the sense that it incorporated residential history, but not time-varying in calendar time as all predictions were estimated for the year 2000.

### Cardiovascular Outcomes

The outcome of interest was time from enrollment until first cardiovascular event, which included myocardial infarction, stroke, death from coronary heart disease, and death from cerebrovascular disease. The WHI identified CVD outcomes through annual follow-up questionnaires to participants. Outcomes were ascertained via local and central review and adjudication of medical records by trained physicians (Curb et al. 2003). Deaths were ascertained via proxy reports and data linkage with the National Death Index of the National Center for Health Statistics. Physician adjudicators reviewed all available records for deaths including hospitalization records, autopsy records, and death certificate diagnoses (Curb et al. 2003). See Supplemental Material, “Women’s Health Initiative Classification Criteria for Cardiovascular Disease Events,” for further details about WHI criteria for classification of CVD events. Institutional review boards at the University of Washington and the Fred Hutchinson Cancer Research Center approved the study.

### Socioeconomic Status

We assessed three distinct SES domains: education, occupation, and income/wealth, which have varied effects on health (Elo 2009). Individual-level SES characteristics were obtained from the baseline questionnaire and included education, family income, and occupation. We included four categories for education (less than high school, high school/GED/trade school, some college/associate degree, and bachelor’s degree or higher), five categories for family income (< $20,000; $20,000–$34,999; $35,000–$49,999; $50,000–$74,999; and ≥ $75,000), and four categories for occupation (managerial/professional, technical/sales/administrative, service/labor, and homemaker).

On the neighborhood level, we had more measures available and included the corresponding measures of percent of adults 25 years and older with a high school degree (education), percent of civilian population 16 years and older with professional/managerial/executive occupations (occupation), median family income (income), and percent of families above the poverty line (income). Data from the 2000 Census was used to assess baseline NSES at the tract level, a unit of geography small enough to be considered a reasonable proxy for neighborhood (Soobader et al. 2006). We also included median home value of owner-occupied housing units as a surrogate of wealth. Income and wealth are not surrogates for one another and both may influence health (Braveman et al. 2005). For instance, wealth may buffer consequences of temporary income loss (e.g., due to unemployment). More importantly, however, wealth may be a better indicator of economic SES among older adults because *a*) income and occupation become less important for retired individuals and *b*) accumulated financial assets such as home ownerships become more significant (Pollack et al. 2007). An individual-level measure of wealth was not available in this data set. Finally, we had available an NSES score that was previously related to incident coronary heart disease in this cohort (Bird et al. 2009). The NSES score is a composite measure of six Census tract-level variables that was created from a confirmatory factor analysis examining 12 theoretically relevant measures and was only available in metropolitan statistical areas (Dubowitz et al. 2008). This index was composed of *a*) percent of adults 25 years and older with less than a high school education, *b*) percent male unemployment, *c*) percent of households with income below the poverty line, *d*) percent of households receiving public assistance, *e*) percent of households with children headed only by a female, and *f*) median household income. Values of the NSES score during intercensal years were interpolated, and participants were assigned baseline values based on their year of enrollment. Higher values on the score indicate less deprivation.

Individual-level SES indicators had weak to moderate correlations with each other and with NSES indicators (correlation coefficients range from 0.14 to 0.36) (see Table S2). NSES indicators exhibited stronger correlation with each other (correlation coefficients from 0.45 to 0.85); even so, NSES indicators represent distinct domains of SES that putatively affect health via distinct mechanisms and pathways (Elo 2009).

### Statistical Analysis

The relationship between long-term annual average PM_2.5_ exposure and time from enrollment until incident cardiovascular events was assessed using Cox proportional hazards models. The following baseline characteristics were controlled for as potential confounders: age, race/ethnicity, diabetes, hypertension, hypercholesterolemia, smoking (smoking status, cigarettes per day, years smoked), and body mass index. Analyses were stratified by 5-year age categories, body mass index (five categories), and diabetes status for a more thorough adjustment. Race/ethnicity was condensed into a binary variable for white not of Hispanic origin and a group including American Indian/Alaskan Native, Asian/Pacific Islander, black, Hispanic, and unknown race/ethnicity due to the small numbers of participants in the latter category.

Although the use of explanatory variables at both the individual and neighborhood levels suggests a multilevel approach, multilevel Cox regression models are often computationally intensive and cumbersome (Goldstein 1995). Therefore, this study utilized the more tractable marginal method which uses traditional estimation. To obtain estimates of standard errors and *p-*values unbiased by geographic clustering of individuals, we adjusted the variances of these coefficients using a sandwich estimator (Lee et al. 1992; Lin 1994).

Effect modification by each individual-level SES and NSES indicator was investigated by fitting multiplicative interaction terms for different levels of the SES variable with PM_2.5_. Separate interaction models were fit for each individual-level SES and NSES variable. Models adjusted for all individual-level SES and NSES variables in addition to other baseline covariates. The model for the composite NSES score included all individual-level SES variables and adjustment covariates but no other NSES variables. Joint tests were conducted to simultaneously test all interaction terms for the SES indicator in question. The Benjamini–Hochberg method was used to control the false discovery rate at 5% (Benjamini and Hochberg 1995). Statistical analyses were performed using Stata (release 13; Stata Statistical Software).

To observe potential confounding by SES variables, we fitted separate models for each SES variable (individual or neighborhood level) and adjusted for all non-SES covariates. Individual-level SES variables were included as categorical variables. All NSES variables were measured continuously, but split into quartiles and included as factor variables in the analyses to allow for non-linear relationships between these measures and time to CVD event. All SES models were adjusted separately (not sequentially). We then proceeded to fit three combinations of SES variables: all individual-level SES only, all NSES only, and both.

### Sensitivity Analysis

Hypertension may lie along the causal pathway between air pollution and CVD. In sensitivity analyses, hypertension was removed from models. We also evaluated whether after adjustment for SES (individual level first and then contextual) there was residual confounding from individual behavioral factors. This is pertinent to large cohort studies using administrative data that lack individual variables. To address the possibility of selection bias due to complete case analysis, missing values in SES variables and adjustment covariates were multiply imputed (see Supplemental Material, “Multiple Imputation”). However, these analyses were run using baseline PM_2.5_ instead of a time-weighted average PM_2.5_ due to issues of computational feasibility. In addition, cross-level interactions were explored, looking at the following categories: low SES in both levels, low SES in one level and high SES in the other, and high SES in both levels (see Supplemental Material, “Cross-level Interaction”).

## Results

Our analytic sample included 51,754 women with 387,840 women-years of follow-up. Mean age at enrollment was 63 years. Most participants were non-Hispanic whites (86.3%) and were never or past smokers (52.4% and 41.4%, respectively) (Table 1). In general, subject characteristics were similar across different categories of first PM_2.5_ prediction, although those in the highest exposure quartile tended to have fewer non-Hispanic whites and lower NSES (Tables 1 and 2). Those who experienced CVD events tended to have less education and lower income, were less likely to work in managerial or professional positions, and more likely to live in lower-NSES neighborhoods (see Table S3).

**Table 1:** Select characteristics of study participants at baseline by quartiles of first PM_2.5_ prediction.

Characteristic	Total mean ± SD or *n* (%)	PM_2.5_ quartile (μg/m^3^)			
		< 10.8 mean ± SD or *n* (%)	10.8–12.4 mean ± SD or *n* (%)	12.5–14.8 mean ± SD or *n* (%)	> 14.9 mean ± SD or *n* (%)
No. of participants**	51,754	12,939	12,938	12,939	12,938
Age (years)	63.0 ± 7.3	63.2 ± 7.2	63.3 ± 7.2	62.9 ± 7.3	62.8 ± 7.3
Race/ethnicity
American Indian/Alaskan Native	193 (0.4)	82 (0.6)	48 (0.4)	34 (0.3)	29 (0.2)
Asian/Pacific Islander	722 (1.4)	160 (1.2)	202 (1.6)	214 (1.7)	146 (1.1)
Black	3,696 (7.1)	222 (1.7)	289 (2.2)	1,038 (8.0)	2,147 (16.6)
Hispanic	2,016 (3.9)	819 (6.3)	427 (3.3)	487 (3.8)	283 (2.2)
White not of Hispanic origin	44,671 (86.3)	11,539 (89.2)	11,863 (91.7)	11,042 (85.3)	10,227 (79.0)
Unknown	456 (0.9)	117 (0.9)	109 (0.8)	124 (1.0)	106 (0.8)
Smoking status
Never smoker	27,102 (52.4)	6,862 (53.0)	6,699 (51.8)	6,651 (51.4)	6,890 (53.3)
Past smoker	21,425 (41.4)	5,343 (41.3)	5,549 (42.9)	5,436 (42.0)	5,097 (39.4)
Current smoker	3,227 (6.2)	734 (5.7)	690 (5.3)	852 (6.6)	951 (7.4)
Body mass index (kg/m^2^)
Normal and underweight (< 25)	21,589 (41.7)	5,412 (41.8)	5,503 (42.5)	5,447 (42.1)	5,227 (40.4)
Overweight (25–29.9)	17,737 (34.3)	4,520 (34.9)	4,441 (34.3)	4,415 (34.1)	4,361 (33.7)
Obese (≥ 30)	12,428 (24.0)	3,007 (23.2)	2,994 (23.1)	3,077 (23.8)	3,350 (25.9)
Hypertension
No	36,553 (70.6)	9,271 (71.7)	9,240 (71.4)	9,209 (71.2)	8,833 (68.3)
Yes	15,201 (29.4)	3,668 (28.3)	3,698 (28.6)	3,730 (28.8)	4,105 (31.7)
Hypercholesterolemia
No	45,335 (87.6)	11,418 (88.2)	11,365 (87.8)	11,318 (87.5)	11,234 (86.8)
Yes	6,419 (12.4)	1,521 (11.8)	1,573 (12.2)	1,621 (12.5)	1,704 (13.2)
Diabetes
No	49,565 (95.8)	12,405 (95.9)	12,469 (96.4)	12,401 (95.8)	12,290 (95.0)
Yes	2,189 (4.2)	534 (4.1)	469 (3.6)	538 (4.2)	648 (5.0)
Note:^******^The first available PM_2.5_ is not the time-weighted average exposure used in models. For most participants, the first available PM_2.5_ prediction was the baseline prediction; otherwise, the next available non-missing PM_2.5_ prediction was used. CVD, cardiovascular disease; PM_2.5_, fine particulate matter.					

**Table 2:** Individual and neighborhood SES characteristics of study participants at baseline by quartiles of first PM_2.5_ prediction.

Characteristic	Total	PM_2.5_ quartile (μg/m^3^)			
		< 10.8 *n* (%)	10.8–12.4 *n* (%)	12.5–14.8 *n* (%)	> 14.9 *n* (%)
No. of participants	51,754	12,939	12,938	12,939	12,938
Individual-level SES
Education
< HS	625 (1.2)	179 (1.4)	109 (0.8)	170 (1.3)	167 (1.3)
HS/trade school/GED	9,873 (19.1)	2,606 (20.1)	2,386 (18.4)	2,362 (18.3)	2,519 (19.5)
Some college or associate degree	4,854 (9.4)	1,275 (9.9)	1,241 (9.6)	1,139 (8.8)	1,199 (9.3)
Bachelor’s degree or higher	36,402 (70.3)	8,879 (68.6)	9,202 (71.1)	9,268 (71.6)	9,053 (70.0)
Family income
< $20,000	1,729 (3.3)	429 (3.3)	327 (2.5)	396 (3.1)	577 (4.5)
$20,000–$34,999	5,219 (10.1)	1,349 (10.4)	1,158 (9.0)	1,272 (9.8)	1,440 (11.1)
$35,000–$49,999	11,428 (22.1)	2,990 (23.1)	2,880 (22.3)	2,739 (21.2)	2,819 (21.8)
$50,000–$74,999	10,373 (20.0)	2,723 (21.0)	2,630 (20.3)	2,415 (18.7)	2,605 (20.1)
$75,000+	23,005 (44.5)	5,448 (42.1)	5,943 (45.9)	6,117 (47.3)	5,497 (42.5)
Occupation at baseline
Managerial/professional	22,796 (44.0)	5,387 (41.6)	5,768 (44.6)	5,950 (46.0)	5,691 (44.0)
Technical/sales/administrative	15,038 (29.1)	3,764 (29.1)	3,836 (29.6)	3,669 (28.4)	3,769 (29.1)
Service/labor	8,583 (16.6)	2,304 (17.8)	2,054 (15.9)	2,041 (15.8)	2,184 (16.9)
Homemaker only	5,337 (10.3)	1,484 (11.5)	1,280 (9.9)	1,279 (9.9)	1,294 (10)
Neighborhood-level SES
Percent of adults 25 years and older with HS degree
< 82.3%	12,952 (25.0)	2,745 (21.2)	2,300 (17.8)	3,666 (28.3)	4,241 (32.8)
82.3–89.4%	12,927 (25.0)	3,470 (26.8)	3,028 (23.4)	3,363 (26.0)	3,066 (23.7)
89.5–94.3%	12,937 (25.0)	3,518 (27.2)	3,610 (27.9)	3,008 (23.2)	2,801 (21.6)
> 94.3%	12,938 (25.0)	3,206 (24.8)	4,000 (30.9)	2,902 (22.4)	2,830 (21.9)
Median family income
< $47,891	12,946 (25.0)	3,188 (24.6)	2,587 (20.0)	2,960 (22.9)	4,211 (32.5)
$47,891–62,526	12,933 (25.0)	3,616 (27.9)	2,956 (22.8)	3,158 (24.4)	3,203 (24.8)
$62,527–81,973	12,939 (25.0)	3,337 (25.8)	3,586 (27.7)	3,122 (24.1)	2,894 (22.4)
> $81,973	12,936 (25.0)	2,798 (21.6)	3,809 (29.4)	3,699 (28.6)	2,630 (20.3)
Percent of civilians 16 years and older with professional/managerial/executive occupations
< 29.7%	12,941 (25.0)	3,251 (25.1)	2,791 (21.6)	3,079 (23.8)	3,820 (29.5)
29.7–41.3%	12,939 (25.0)	3,843 (29.7)	3,083 (23.8)	3,059 (23.6)	2,954 (22.8)
41.4–54.1%	12,939 (25.0)	3,313 (25.6)	3,442 (26.6)	3,179 (24.6)	3,005 (23.2)
> 54.1%	12,935 (25.0)	2,532 (19.6)	3,622 (28)	3,622 (28.0)	3,159 (24.4)
Median home value
< $103,500	12,939 (25.0)	2,755 (21.3)	2,764 (21.4)	3,224 (24.9)	4,196 (32.4)
$103,500–153,599	12,943 (25.0)	3,615 (27.9)	3,484 (26.9)	2,375 (18.4)	3,469 (26.8)
$153,600–233,999	12,934 (25.0)	3,511 (27.1)	2,857 (22.1)	3,594 (27.8)	2,972 (23.0)
> $233,999	12,938 (25.0)	3,058 (23.6)	3,833 (29.6)	3,746 (29)	2,301 (17.8)
Percent of families above poverty line
< 89.2%	12,940 (25.0)	2,729 (21.1)	2,573 (19.9)	3,146 (24.3)	4,492 (34.7)
89.2–94.0%	12,940 (25.0)	3,413 (26.4)	3,011 (23.3)	3,424 (26.5)	3,092 (23.9)
94.1–96.5%	12,943 (25.0)	3,711 (28.7)	3,361 (26)	3,060 (23.6)	2,811 (21.7)
> 96.5%	12,931 (25.0)	3,086 (23.9)	3,993 (30.9)	3,309 (25.6)	2,543 (19.7)
NSES score
< 72.6	12,939 (25.0)	2,791 (21.6)	2,492 (19.3)	3,211 (24.8)	4,445 (34.4)
72.6–77.6	12,938 (25.0)	3,750 (29.0)	2,937 (22.7)	3,352 (25.9)	2,899 (22.4)
77.7–81.6	12,939 (25.0)	3,487 (26.9)	3,445 (26.6)	3,020 (23.3)	2,987 (23.1)
> 81.6	12,938 (25.0)	2,911 (22.5)	4,064 (31.4)	3,356 (25.9)	2,607 (20.1)
Note: The first available PM_2.5_ is not the time-weighted average exposure used in models. For most participants, the first available PM_2.5_ prediction was the baseline prediction; otherwise, the next available non-missing PM_2.5_ prediction was used. CVD, cardiovascular disease; HS, high school; NSES, neighborhood socioeconomic status; PM_2.5_, fine particulate matter; SES, socioeconomic status.					

We observed 1,737 cardiovascular events. The number of events in each quartile of first available PM_2.5_ prediction is shown in Table 3. The highest number of events was observed in the highest quartile of PM_2.5_. The overall mean concentration of all PM_2.5_ observations was 12.7 μg/m^3^ (SD, 2.9; interquartile range, 4.1); the minimum was 2.2 μg/m^3^ and the maximum was 25.1 μg/m^3^. Figure S1 shows a scatterplot of first available PM_2.5_ predictions by NSES score with a locally weighted scatterplot smoothing curve. In general, areas with lower NSES tended to experience slightly higher levels of PM_2.5_.

**Table 3:** Number of cardiovascular events by quartiles of first PM_2.5_ prediction.

No. of participants or events	Total	Quartiles of PM_2.5_ (μg/m^3^)			
		< 10.8 *n* (%)	10.8–12.4 *n* (%)	12.5–14.8 *n* (%)	> 14.9 *n* (%)
No. of participants	51,754	12,939	12,938	12,939	12,938
No. of events	1,737 (3.4)	415 (3.2)	431 (3.3)	398 (3.1)	493 (3.8)
Note: The first available PM_2.5_ is not the time-weighted average exposure used in models. For most participants, the first available PM_2.5_ prediction was the baseline prediction; otherwise, the next available non-missing PM_2.5_ prediction was used. PM_2.5_, fine particulate matter.					

Exposure to PM_2.5_ was significantly associated with risk of cardiovascular events. After adjustment for age, race/ethnicity, smoking, body mass index, diabetes, hypertension, and hypercholesterolemia, a 5 μg/m^3^ higher exposure to PM_2.5_ was associated with a 12% higher risk of cardiovascular event [hazard ratio (HR): 1.12; 95% confidence interval (CI): 1.00, 1.25; Table 4]. Further adjustment for individual-level SES or NSES (singly or combined) did not change the HR materially. In Table 4, each adjustment listed is separate and not sequentially related to the adjustment above it. For example, the individual income model only adjusted for individual income and all non-SES covariates. The fully adjusted model, which included all potential confounders and all individual-level SES and NSES variables except for the NSES score, had an HR of 1.13 (95% CI: 1.02, 1.25).

**Table 4:** Estimated hazard ratios for time to first cardiovascular event associated with 5 μg/m^3^ higher exposure to PM_2.5_, with additional adjustment for each socioeconomic measure.

Characteristic	HR (95% CI)
PM_2.5_ without SES measures	1.12 (1.00, 1.25)
Individual-level SES
Education	1.12 (1.01, 1.25)
Income	1.12 (1.01, 1.24)
Occupation	1.12 (1.01, 1.25)
All Individual-level SES^*a*^	1.12 (1.01, 1.25)
NSES
Education	1.12 (1.00, 1.25)
Income	1.12 (1.00, 1.24)
Employment	1.12 (1.00, 1.25)
Home values	1.12 (1.00, 1.24)
Poverty	1.12 (1.01, 1.25)
NSES score	1.12 (1.00, 1.25)
All NSES (no NSES score)^*b*^	1.13 (1.01, 1.25)
Individual-level SES and NSES
All individual-level SES and NSES score^*c*^	1.13 (1.02, 1.26)
All individual-level SES and All NSES (no NSES score)^*d*^	1.13 (1.02, 1.25)
Note: All hazard ratios (HR) are adjusted for age, race/ethnicity, smoking, body mass index, diabetes, hypertension, and hypercholesterolemia. Models listed are separate from one another and are not sequentially adjusted. The models adjust for SES measures indicated and no other SES measures listed above or below it. Models adjusting for combinations of SES measures (e.g., All Individual-level SES) are notated and explained in footnotes *a*–*d*. CI, confidence interval; HR, hazard ratio; NSES, neighborhood-level SES status; PM_2.5_, fine particulate matter; SES, socioeconomic status. ^***a***^Additionally adjusted for the following individual-level SES indicators: education, total family income, and occupation. ^***b***^Additionally adjusted for the following NSES indicators: percent of adults 25 years and older with high school degree, median family income, percent of civilian population 16 years and older with professional/managerial/executive occupations, median value of owner-occupied housing units, and percent of families above poverty line. This model does not include the NSES score. ^***c***^Additionally adjusted for the NSES score and all individual-level SES indicators. ^***d***^Additionally adjusted for all individual-level SES and NSES indicators except for NSES score.	

**Figure 1:**
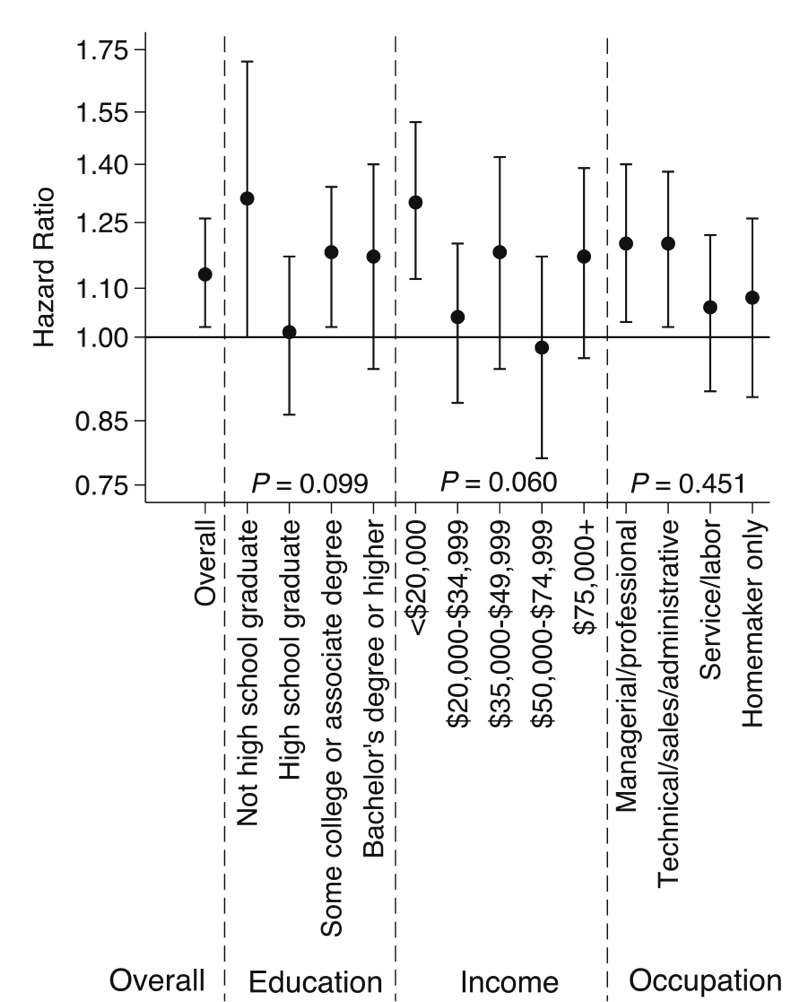
Estimated hazard ratios and 95% confidence intervals for time to first cardiovascular event associated with 5 μg/m^3^ higher exposure to PM_2.5_ according to levels of individual socioeconomic status (SES) and *p*-values for interactions. Models adjusted for age, race/ethnicity, smoking, body mass index, diabetes, hypertension, hypercholesterolemia, and all individual-level SES and neighborhood-level SES indicators except for the neighborhood SES score.

**Figure 2:**
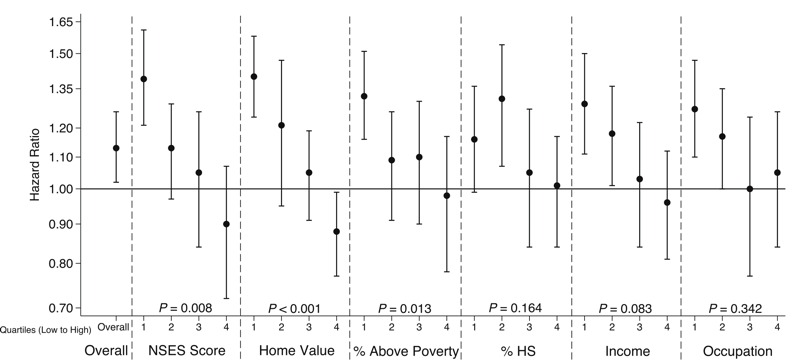
Estimated hazard ratios and 95% confidence intervals for time to first cardiovascular event associated with 5 μg/m^3^ higher exposure to PM_2.5_ according to levels of neighborhood socioeconomic status (NSES) and *p*-values for interactions. Models adjusted for age, race/ethnicity, smoking, body mass index, diabetes, hypertension, hypercholesterolemia, and all individual-level socioeconomic status and NSES indicators except for the NSES score. All NSES variables were grouped into quartiles, ranging from lowest NSES (most deprived) to highest NSES (least deprived). The NSES score model adjusted for individual-level SES indicators but no other NSES indicators.

The associations of PM_2.5_ with CVD events by categories of individual SES and NSES variables are shown in Figures 1 and 2, respectively. None of the individual-level SES variables significantly modified the association between PM_2.5_ and CVD events. Although those with the lowest individual income (< $20,000) had an HR of 1.30 (95% CI: 1.12, 1.52), it was not significantly different from other income categories. There is evidence of statistically significant effect modification by the NSES score (2-sided *p* = 0.008), median home value (2-sided *p* < 0.001), and percentage above poverty (2-sided *p* = 0.013) after accounting for multiple comparisons. Those in the most disadvantaged quartile of the NSES score had an HR of 1.39 (95% CI: 1.21, 1.61); whereas, those in the least disadvantaged quartile had an HR of 0.90 (95% CI: 0.72, 1.07). Similarly, those in the lowest quartile of median home value had an HR of 1.40 (95% CI: 1.24, 1.58) compared to those in the highest quartile with an HR of 0.87 (95% CI: 0.77, 0.99). Furthermore, significant positive associations between PM_2.5_ exposure and CVD risk were observed in the most disadvantaged quartiles of all NSES variables examined. HRs tended to decrease as NSES increased, and this trend is consistent across categories for multiple neighborhood-level indicators, unlike for individual-level SES indicators.

In sensitivity analyses, removal of hypertension did not change our results materially (see Table S4). In the model only adjusted for age, the estimated HR was 1.13 (95% CI: 1.01, 1.28). Additional control for SES indicators in age-only models did not change HRs meaningfully. There was no evidence of confounding by SES indicators even without controlling for individual risk factors and no indication of residual confounding by individual factors. Multiple imputation of missing covariates, including SES variables, did not change the results materially (see Tables S5 and S6). Looking at cross-level interactions, having both low individual education and low NSES (any characteristic) did not confer greater vulnerability than having low SES on only one level (see Figure S2). However, there is evidence that having low individual income and low NSES (any characteristic) conferred greater risk of CVD than having high SES on at least one level.

## Discussion

Results corroborate previous studies that exposure to long-term PM_2.5_ is a risk factor for CVD, and this association cannot be explained by confounding by individual-level SES or NSES. Furthermore, the association was stronger for women residing in lower-SES neighborhoods.

Our results of effect modification by NSES are consistent with the hypothesis that those with low SES may be disproportionately affected by the adverse health effects of air pollution. Researchers documented that individuals with low SES and racial minorities experience higher exposure to air pollution (Hajat et al. 2015) and also suffer from worse health outcomes resulting from poverty and psychosocial stress in poor communities (Diez Roux et al. 2004). The combination of greater exposure to air pollution, poorer health, and fewer resources to cope with the effects of air pollution may result in increased susceptibility to air pollution-related health outcomes (O’Neill et al. 2003). We see stronger effect modification for neighborhood property values compared to median household income, which might stem from property values being a better reflection of SES for older individuals. In addition, it is possible that property values may be higher in communities of more owners (vs. renters), and these owners may be more invested in the long term, which could contribute to contextual factors such as neighborhood stability or investment, and other social processes not captured by income or administrative data.

The lack of evidence for effect modification by individual-level SES suggests that neighborhood-level processes may increase susceptibility to air pollution-related CVD. First, NSES is on the same spatial scale as air pollution, and empirical evidence shows that the association between individual-level SES and PM_2.5_ is often weaker than that observed between NSES and air pollution (Hajat et al. 2013). Secondly, macro-level contextual factors, such as racial-residential segregation are hypothesized to differentially distribute exposures to environmental hazards and to concentrate poverty (Gee and Payne-Sturges 2004; Morello-Frosch and Lopez 2006). Disadvantaged neighborhood environments may be working through the stress pathway to impact health (Diez Roux and Mair 2010) making residents more susceptible to the health effects of PM_2.5_.

Exposure measurement error, particulate matter infiltration, dose reduction, and subject time activity patterns may differ according to individual-level SES or NSES and could explain part of our findings of effect modification by NSES. Higher rates of PM_2.5_ infiltration have been reported for lower-SES individuals (Hystad et al. 2009), which may be explained by decreased use of air conditioning and older and poorer housing quality among low-SES individuals. Thus, using ambient exposures would systematically underestimate true exposures for lower-SES persons compared to those with higher SES, which would be consistent with the direction of effect modification observed in this study. Furthermore, the health-motivated individuals among those with more resources may use their resources not only to seek cleaner residential areas but also to reduce background exposures (e.g., by better air conditioning). Thus, some of the effect modification may actually represent true dose reduction in those with high NSES.

Our findings of no confounding by individual-level SES in this cohort are consistent with studies reporting small changes in relative risk estimates after adjustment for SES including education and income (Brochu et al. 2011; Dockery et al. 1993; Pope et al. 2002). However, research in Canada suggested that NSES positively confounded the relationship between particulate air pollution and mortality, where adjustment for several NSES variables changed risk estimates more than 10% (Jerrett et al. 2005). The WHI cohort has higher SES relative to the United States as a whole, and relatively small SES variability in our data could explain the lack of confounding by SES in our results. In addition, while individual-level SES is associated with CVD outcomes, it is not strongly associated with exposure, and the converse is true for NSES (NSES is associated with exposure but not strongly associated with CVD outcomes)—hence, neither served as strong confounders. Either individual-level SES or NSES may still be an important confounder in populations where associations between SES and air pollution and SES and CVD are large.

NSES may also lie along the causal pathway between air pollution and CVD. For example, poor air quality due to a polluting facility and increasing traffic may change the attractiveness of a neighborhood, causing higher SES individuals to move away, lower-SES individuals to move in, and/or home values to decline. The resulting lower NSES of the neighborhood may cause negative changes in the neighborhood’s social, physical, and built environments that could result in adverse health effects for residents. If so, NSES would be in the causal pathway and should be dealt with in an analytically appropriate manner. Understanding the directionality of NSES and air quality is difficult especially in a multisite study of this nature where different processes are likely occurring in different places (Saha and Mohai 2005).

This study is consistent with the findings in the analysis by Miller et al. (HR = 1.24; 95% CI: 1.09, 1.4; per 10 μg/m^3^ higher PM_2.5_) (Miller et al. 2007) and analysis in a Health Effects Institute report (HR = 1.25; 95% CI: 1.09, 1.44; per 10 μg/m^3^ higher PM_2.5_) (Vedal et al. 2013) which used the same cohort of women. Our fully adjusted HR is 1.29 (95% CI: 1.04, 1.59) when scaled to a 10 μg/m^3^ higher exposure to PM_2.5._ However, the current analysis is primarily focused (unlike the prior analyses) on the very important methodological problem of disentangling air pollution exposures from putatively co-varying socioeconomic factors. The first study (Miller et al. 2007) assessed NSES in a sensitivity analysis and found no evidence of confounding by U.S. Census-derived measures aggregated to ZIP code level, but did not assess NSES as a potential effect modifier. The current study also uses improved exposure assessment that is resolved to participant addresses and incorporates residential history. The first study assigned exposures based on nearest monitor to participant homes. The second utilized baseline PM_2.5_ predictions at geocoded addresses, but included sensitivity analyses that incorporated exposures based on residential location in the 1 or 2 years before event or death (or corresponding years in subjects with no events). The current study uses a time-weighted average PM_2.5_ that incorporates all residential history. Finally, the current study had 2 additional years of follow-up compared to the first study.

There are several limitations to this study. First, selection bias may arise from conducting a complete case analysis. However, important measures including exposure and CVD incidence were well-balanced between complete cases and eligible participants in the original sample (see Table S1), indicating that our analytic sample is generally representative of the baseline sample of women who were CVD free. There was also no individual measure of wealth available, and we were not able to assess either effect modification or confounding by individual wealth. In addition, sensitivity analyses that multiply imputed all SES variables and adjustment covariates were not materially different from the main analysis. Another issue is unobserved confounding by self-selection into neighborhoods. However, our estimates are robust to adjustment for many demographic, socioeconomic, and health characteristics that may correlate with self-selection. We also do not have measures of indoor air pollution, which may better reflect true exposures in an older population which spends more time indoors. Future studies are needed to assess the effect of this measurement error. Our SES measures were not adjusted for variation in cost of living and housing in different regions, which may lead to measurement error in our SES assessment in a national cohort. The effect of this error is likely to be location- and population-specific. However, while developing the NSES score, the authors conducted sensitivity analysis to adjust for differences in cost of living, and it did not have an effect on the NSES score. Further research is needed to assess the importance of adjusting for cost of living in the measurement of individual-level SES. Future work is also needed to test for sex differences and the impact of NSES on the association between PM_2.5_ and CVD events in samples that are more representative of the United States.

This study has several strengths. First, analyses were conducted using a large sample size and a long follow-up time. Second, outcomes were adjudicated based on protocol-based review of medical records, thereby reducing outcome misclassification. Third, we were able to resolve PM_2.5_ exposures to the level of the individual’s residence based on geocodes and a state-of-the-art fine-scale modeling framework, reducing exposure misclassification. In addition, this study is among the few to investigate the roles of both individual-level SES and NSES in different domains on the association between air pollution and cardiovascular disease, which is an important methodological improvement on prior attempts to measure SES. Finally, the study examines a range of women of moderate income residing across a wide range of NSES, which gives considerable ability to assess the impact of NSES. Many past studies did not have data on both.

## Conclusion

We investigated the role that SES plays in the association between PM_2.5_ and CVD. We found that individual-level SES and NSES did not confound the positive association between PM_2.5_ and CVD in this cohort. Furthermore, risk estimates were higher for women living in more disadvantaged neighborhoods. Our findings contribute to the understanding of confounding by SES in air pollution health effects research and support an evolving understanding of the synergistic adverse effects of air pollution and socioeconomic factors.

## Supplemental Material

(816 KB) PDF
