# Supplementary material for: Individual and Neighborhood Socioeconomic Status and the Association between Air Pollution and Cardiovascular Disease
Source: Environ Health Perspect. 2016 May 3;124(12):1840–7. doi: 10.1289/EHP199 (PMC5132637; doi:10.1289/EHP199)
Supplement: (816 KB) PDF [file EHP199.s001.acco.pdf]

Environ Health Perspect
DOI: 10.1289/EHP199

Note to readers with disabilities: *EHP* strives to ensure that all journal content is accessible to all readers. However, some figures and Supplemental Material published in *EHP* articles may not conform to 508 standards due to the complexity of the information being presented. If you need assistance accessing journal content, please contact ehp508@niehs.nih.gov. Our staff will work with you to assess and meet your accessibility needs within 3 working days.

Supplemental Material

Individual and Neighborhood Socioeconomic Status and the Association between Air Pollution and Cardiovascular Disease

Gloria C. Chi, Anjum Hajat, Chloe E. Bird, Mark R. Cullen, Beth Ann Griffin, Kristin A. Miller, Regina A. Shih, Marcia L. Stefanick, Sverre Vedal, Eric A. Whitsel, and Joel D. Kaufman

Table of Contents

Women's Health Initiative Classification Criteria for Cardiovascular Disease Events
Cross-level Interaction
Multiple Imputation
Table S1. Standardized mean differences of participant characteristics between complete cases and original eligible sample of women CVD-free at baseline.
Table S2. Correlation Matrix of Socioeconomic Indicators.
Table S3. Baseline population characteristics by cardiovascular event incidence.
Table S4. Estimated hazard ratios for time to first cardiovascular event associated with 5 μg/m^3 higher exposure to $PM_{2.5}$, by different levels of covariate adjustment and with additional adjustment for each socioeconomic measure.
Table S5. Estimated hazard ratios for time to first cardiovascular event associated with 5 μg/m^3 higher exposure to baseline $PM_{2.5}$ from multiple imputation analysis.

Table S6. Estimated hazard ratios for time to first cardiovascular event associated with 5 μg/m^3 higher exposure to $PM_{2.5}$ according to levels of SES and *p*-values for interactions, from multiple imputation analysis.

Figure S1. Scatter plot of first predicted annual average $PM_{2.5}$ by neighborhood socioeconomic status (NSES) score with a locally weighted scatterplot smoothing (LOWESS) curve. For most participants, the first available $PM_{2.5}$ prediction was the baseline prediction; otherwise, the next available non-missing $PM_{2.5}$ prediction was used.

Figure S2. Estimated Hazard Ratios for Time to First Cardiovascular Event Associated with 5 μg/m^3 Higher Exposure to Fine Particulate Matter ($PM_{2.5}$) According to Combined Levels of Individual and Neighborhood Socioeconomic Status (SES) and *P* values for interactions, Women's Health Initiative Observational Study, 1993-2005. All models except for the NSES score model (see below) adjusted for age, race/ethnicity, smoking, body mass index, diabetes, hypertension, hypercholesterolemia, individual education, individual family income, individual occupation, percent adults over 25 years with high school degree, median family income, percent of civilian population over 16 with professional/managerial/executive occupations, median value of owner-occupied housing units, and percent of families above poverty line. Effect modification was modeled by adding multiplicative interaction terms. The NSES score model adjusted for individual SES indicators but not other NSES indicators. A) Estimated hazard ratios by levels of combined NSES and individual education. Low/Low participants have low individual education and low neighborhood SES (NSES). Low/High participants have low education and high NSES or high education and low NSES. High/High participants have high education and high NSES. B) Estimated hazard ratios by levels of combined NSES and individual income. Low/Low participants have low individual income and low NSES. Low/High participants have low income and high NSES or high income and low NSES. High/High participants have high income and high NSES.

Women's Health Initiative classification criteria for cardiovascular disease events

The outcome of interest in this study included time to first cardiovascular event (defined as myocardial infarction (MI), stroke, and death from coronary heart disease (CHD) or cerebrovascular disease). An algorithm that incorporated elements of the medical history, electrocardiogram readings, and results of cardiac enzyme/troponin determinations were used to identify and categorize MI's into definite or possible MI's.[21] Strokes included persistent neurological deficit of more than 24 hour duration, due to obstruction or rupture in the brain arterial system. Deaths due to coronary heart disease include deaths due to definite MI's and to definite or possible CHD. Definite fatal CHD were those with no known non-atherosclerotic cause and chest pain within the 72 hours of death and/or a history of chronic disease (with no valvular heart disease or non-ischemic cardiomyopathy). Possible atherosclerotic cardiac disease death was defined as those with no known non-atherosclerotic cause that were consistent with CHD as an underlying cause.

Cross-level interaction

Cross-level interactions were explored in sensitivity analyses. On the individual level, participant education (HS/trade school/GED or less vs. some college or more) and income (<$50,000 vs. $50,000 or more) were dichotomized. All neighborhood socioeconomic status (NSES) characteristics were dichotomized at the median. For each NSES characteristic and individual-level education or income, participants were categorized as low on both levels (low/low), low on one level and high on the other (low/high), and high on both levels. We were unable to separate out those who had discordant socioeconomic status (SES) levels as low individual SES/high NSES or vice versa due to small numbers. The relationship between long-term annual average $PM_{2.5}$ exposure and time until incident cardiovascular events was assessed using Cox proportional hazards models. Potential effect modification by SES was investigated by fitting multiplicative interaction terms for different levels of the SES variable with $PM_{2.5}$. All models except for the NSES score models adjusted for age,

race/ethnicity, smoking, body mass index, diabetes, hypertension, hypercholesterolemia, individual education, individual family income, individual occupation, percent adults over 25 years with high school degree, median family income, percent of civilian population over 16 with professional/managerial/executive occupations, median value of owner-occupied housing units, and percent of families above poverty line. The NSES score model adjusted for all covariates except for other neighborhood-level SES indicators.

Results for the cross-level interactions can be found in Figure S1. There was no evidence that having low education and low NSES conferred greater disadvantage than having low SES on only one level. However, there is evidence that the association between $PM_{2.5}$ and CVD is the greatest among those with low income and low NSES, even more so than those who have low SES on only one level.

Multiple imputation

Multiple imputation analyses included 60,744 women who did not have CVD at baseline and did not have missing baseline $PM_{2.5}$ predictions. These analyses were conducted using baseline $PM_{2.5}$ instead of time-weighted $PM_{2.5}$ for computational feasibility. Missing values in all SES indicators and covariates were multiply imputed 5 times using chained equations in Stata 13 (StataCorp 2013). The relationship between long-term annual average $PM_{2.5}$ exposure and time from enrollment until incident cardiovascular events was assessed using Cox proportional hazards models. The same baseline characteristics were controlled for as potential confounders as in the main analysis: age, race/ethnicity, diabetes, hypertension, hypercholesterolemia, smoking (smoking status, cigarettes per day, years smoked), and body mass index. Analyses were stratified by 5 year age categories, body mass index (5 categories), and diabetes status for a more thorough adjustment.

Table S1. Standardized mean differences of participant characteristics between complete cases and original eligible sample of women CVD-free at baseline.

	Original Eligible Sample of CVD-Free Women at Baseline N=73,094		Complete Case N=51,754		Standardized Mean Difference
Variable	Mean	SD	Mean	SD	
$PM_{2.5}$ prediction	12.743	2.946	12.650	2.914	0.032
CVD event	0.034	0.181	0.034	0.180	0.001
Age	63.086	7.304	63.037	7.273	0.007
Race/ethnicity					
American Indian/Alaskan Native	0.004	0.063	0.004	0.061	0.005
Asian/Pacific Islander	0.031	0.174	0.014	0.117	0.099
Black	0.078	0.268	0.071	0.258	0.025
Hispanic	0.039	0.195	0.039	0.193	0.003
White not of Hispanic origin	0.834	0.372	0.863	0.344	-0.079
Unknown	0.011	0.104	0.009	0.093	0.02
Body mass index	27.081	5.740	27.105	5.714	-0.004
Percent adults 25+ with HS degree	0.865	0.108	0.866	0.107	-0.013
Percent civilians over 16 with professional/managerial/executive occupations	0.423	0.160	0.424	0.160	-0.011
Median family income	68,056.312	29,549.337	68,139.614	29,401.450	-0.003
Median home value	200,541.217	148,648.707	195,228.912	145,024.650	0.036
Percent families above poverty line	0.912	0.082	0.914	0.081	-0.015
NSES score	76.136	8.495	76.298	8.406	-0.019
Hypertension					
No	0.701	0.458	0.706	0.455	-0.011
Yes	0.294	0.456	0.294	0.455	0.001
Diabetes					
No	0.955	0.208	0.958	0.201	-0.015

Yes	0.045	0.207	0.042	0.201	0.012
Smoking status					
Never smoker	0.508	0.500	0.524	0.499	-0.031
Past smoker	0.419	0.493	0.414	0.493	0.010
Current smoker	0.061	0.239	0.062	0.242	-0.007
Hypercholesterolemia					
No	0.866	0.341	0.876	0.330	-0.030
Yes	0.124	0.330	0.124	0.330	0.001
Education					
Less than HS	0.047	0.211	0.041	0.199	0.027
HS/trade school/GED	0.254	0.435	0.255	0.436	-0.004
Some college or associate degree	0.262	0.440	0.264	0.441	-0.004
Bachelor's degree or higher	0.429	0.495	0.440	0.496	-0.021
Family income					
<$20,000	0.134	0.341	0.134	0.341	-0.001
$20,000-$34,999	0.210	0.407	0.221	0.415	-0.027
$35,000-$49,999	0.189	0.391	0.200	0.400	-0.030
$50,000-$74,999	0.194	0.395	0.203	0.403	-0.025
$75,000+	0.231	0.421	0.241	0.428	-0.025
Occupation at baseline					
Managerial/professional	0.423	0.494	0.440	0.496	-0.036
Technical/sales/administrative	0.276	0.447	0.291	0.454	-0.033
Service/labor	0.162	0.369	0.166	0.372	-0.010
Homemaker only	0.102	0.302	0.103	0.304	-0.005

Table S2. Correlation Matrix of Socioeconomic Indicators.

	Individual SES[a]		Neighborhood SES					
	Education	Income	Education	Income	Employment	Home Values	Poverty	NSES Score
Individual SES								
Education	1							
Income	0.3619	1						
Neighborhood SES								
Education	0.2735	0.3053	1					
Income	0.2656	0.3524	0.763	1				
Employment	0.3187	0.3307	0.7946	0.7909	1			
Home values	0.2485	0.2941	0.5815	0.7493	0.6729	1		
Poverty	0.1374	0.2462	0.6003	0.6984	0.5008	0.4485	1	
NSES score	0.2196	0.3218	0.7722	0.8542	0.7057	0.6202	0.7966	1

SES, socioeconomic status; NSES, neighborhood socioeconomic status.

[a] Individual occupation was included as the following categories (managerial/professional, technical/sales/administrative, service/labor, and homemaker) and thus not included in the correlation matrix.

Table S3. Baseline population characteristics by cardiovascular event incidence.

	Total	CVD Events	
		Yes	No
	N (%)	N (%)	N (%)
Race/ethnicity			
American Indian/Alaskan Native	193 (0.4)	8 (0.5)	185 (0.4)
Asian/Pacific Islander	722 (1.4)	20 (1.2)	702 (1.4)
Black	3,696 (7.1)	127 (7.3)	3,569 (7.1)
Hispanic	2,016 (3.9)	35 (2.0)	1,981 (3.9)
White not of Hispanic origin	44,671 (86.3)	1,529 (88.0)	43,142 (86.3)
Unknown	456 (0.9)	18 (1.0)	438 (0.9)
Smoking status			
Never smoker	27,102 (52.4)	864 (49.7)	26,238 (52.4)
Past smoker	21,425 (41.4)	727 (41.9)	20,698 (41.4)
Current smoker	3,227 (6.2)	146 (8.4)	3,081 (6.2)
Body Mass Index			
Normal and underweight (<25)	21,589 (41.7)	616 (35.5)	20,973 (41.7)
Overweight (25-29.9)	17,737 (34.3)	629 (36.2)	17,108 (34.3)
Obese (>=30)	12,428 (24.0)	492 (28.3)	11,936 (24.0)
Hypertension			
No	36,553 (70.6)	874 (50.3)	35,679 (70.6)
Yes	15,201 (29.4)	863 (49.7)	14,338 (29.4)
Hypercholesterolemia			
No	45,335 (87.6)	1,471 (84.7)	43,864 (87.6)
Yes	6,419 (12.4)	266 (15.3)	6,153 (12.4)
Diabetes			
No	49,565 (95.8)	1,545 (88.9)	48,020 (95.8)
Yes	2,189 (4.2)	192 (11.1)	1,997 (4.2)
**Individual SES**			
Education			
Less than HS	2,135 (4.1)	105 (6.0)	2,030 (4.1)
HS/trade school/GED	13,217 (25.5)	546 (31.4)	12,671 (25.5)
Some college or associate degree	13,647 (26.4)	477 (27.5)	13,170 (26.4)
Bachelor's degree or higher	22,755 (44.0)	609 (35.1)	22,146 (44.0)
Family income			
<$20,000	6,948 (13.4)	358 (20.6)	6,590 (13.4)
$20,000-$34,999	11,428 (22.1)	499 (28.7)	10,929 (22.1)
$35,000-$49,999	10,373 (20.0)	340 (19.6)	10,033 (20.0)
$50,000-$74,999	10,531 (20.3)	255 (14.7)	10,276 (20.3)
$75,000+	12,474 (24.1)	285 (16.4)	12,189 (24.1)
Occupation at baseline			
Managerial/professional	22,796 (44.0)	644 (37.1)	22,152 (44.0)
Technical/sales/administrative	15,038 (29.1)	541 (31.1)	14,497 (29.1)
Service/labor	8,583 (16.6)	327 (18.8)	8,256 (16.6)

Homemaker only	5,337 (10.3)	225 (13.0)	5,112 (10.3)
**Neighborhood SES**			
Percent adults 25+ with HS degree			
<82.3%	12,952 (25.0)	461 (26.5)	12,491 (25.0)
82.3-89.4%	12,927 (25.0)	438 (25.2)	12,489 (25.0)
89.5-94.3%+	12,937 (25.0)	413 (23.8)	12,524 (25.0)
>94.3%	12,938 (25.0)	425 (24.5)	12,513 (25.0)
Median family income			
<$47,891	12,946 (25.0)	491 (28.3)	12,455 (25.0)
$47,891-62,526	12,933 (25.0)	421 (24.2)	12,512 (25.0)
$62,527-81,973	12,939 (25.0)	430 (24.8)	12,509 (25.0)
>$81,973	12,936 (25.0)	395 (22.7)	12,541 (25.0)
Percent civilians over 16 with professional/managerial/executive occupations			
<29.7%	12,941 (25.0)	461 (26.5)	12,480 (25.0)
29.7-41.3%	12,939 (25.0)	429 (24.7)	12,510 (25.0)
41.4-54.1%	12,939 (25.0)	441 (25.4)	12,498 (25.0)
>54.1%	12,935 (25.0)	406 (23.4)	12,529 (25.0)
Median home value			
<$103,500	12,939 (25.0)	482 (27.7)	12,457 (25.0)
$103,500-153,599	12,943 (25.0)	450 (25.9)	12,493 (25.0)
$153,600-233,999	12,934 (25.0)	385 (22.2)	12,549 (25.0)
>$233,999	12,938 (25.0)	420 (24.2)	12,518 (25.0)
Percent families above poverty line			
<89.2%	12,940 (25.0)	453 (26.1)	12,487 (25.0)
89.2-94.0%	12,940 (25.0)	457 (26.3)	12,483 (25.0)
94.1-96.5%	12,943 (25.0)	433 (24.9)	12,510 (25.0)
>96.5%	12,931 (25.0)	394 (22.7)	12,537 (25.0)
NSES score			
<72.6	12,939 (25.0)	465 (26.8)	12,474 (25.0)
72.6-77.6	12,938 (25.0)	450 (25.9)	12,488 (25.0)
77.7-81.6	12,939 (25.0)	426 (24.5)	12,513 (25.0)
>81.6	12,938 (25.0)	396 (22.8)	12,542 (25.0)

$PM_{2.5}$, fine particulate matter; CVD, cardiovascular disease; SES, socioeconomic status; NSES, neighborhood socioeconomic status; HS, high school.

Table S4. Estimated hazard ratios for time to first cardiovascular event associated with 5 μg/m^3 higher exposure to $PM_{2.5}$, by different levels of covariate adjustment and with additional adjustment for each socioeconomic measure.[a]

	All Covariates[b]		No Hypertension[c]		Age (no other non-SES confounders)[d]	
	HR	(95% CI)	HR	(95% CI)	HR	(95% CI)
$PM_{2.5}$ without SES measures	1.12	(1.00, 1.25)	1.12	(1.00, 1.26)	1.13	(1.01, 1.28)
Individual SES						
Education	1.12	(1.01, 1.25)	1.13	(1.01, 1.26)	1.14	(1.02, 1.27)
Income	1.12	(1.01, 1.24)	1.12	(1.01, 1.26)	1.13	(1.01, 1.26)
Occupation	1.12	(1.01, 1.25)	1.12	(1.00, 1.26)	1.13	(1.01, 1.27)
All Individual SES[e]	1.12	(1.01, 1.25)	1.13	(1.01, 1.26)	1.13	(1.02, 1.26)
NSES						
Education	1.12	(1.00, 1.25)	1.12	(1.00, 1.26)	1.12	(0.99, 1.26)
Income	1.12	(1.00, 1.24)	1.12	(1.00, 1.26)	1.12	(1.00, 1.26)
Employment	1.12	(1.00, 1.25)	1.12	(1.00, 1.26)	1.13	(1.01, 1.27)
Home values	1.12	(1.00, 1.24)	1.12	(1.00, 1.26)	1.12	(1.00, 1.26)
Poverty	1.12	(1.01, 1.25)	1.13	(1.01, 1.27)	1.13	(1.00, 1.27)
NSES score	1.12	(1.00, 1.25)	1.12	(1.00, 1.26)	1.13	(1.00, 1.27)
All NSES (no NSES score)[f]	1.13	(1.01, 1.25)	1.13	(1.01, 1.27)	1.13	(1.01, 1.27)
Individual SES and NSES						
All Individual SES + NSES score[g]	1.13	(1.02, 1.25)	1.13	(1.01, 1.27)	1.13	(1.02, 1.27)
All Individual SES + All NSES (no NSES score)[h]	1.13	(1.02, 1.26)	1.14	(1.02, 1.27)	1.14	(1.02, 1.27)

$PM_{2.5}$, fine particulate matter; HR, hazard ratio; CI, confidence interval; SES, socioeconomic status; NSES, neighborhood socioeconomic status.

[a] Models listed are separate from one another and are not sequentially adjusted. The models adjust for SES measures indicated and no other SES measures listed above or below it. Models adjusting for combinations of SES measures (i.e. All Individual SES) are notated and explained below.

[b] HRs are adjusted for age, race/ethnicity, smoking, body mass index, diabetes, hypertension, and hypercholesterolemia.

[c] HRs are adjusted for age, race/ethnicity, smoking, body mass index, diabetes, and hypercholesterolemia.

[d] HRs adjusted for age and no other non-SES confounders.

[e] Additionally adjusted for the following individual-level SES indicators: education, total family income, and occupation.

[f] Additionally adjusted for the following neighborhood-level SES indicators: percent adults over 25 years with high school degree, median family income, percent of civilian population over 16 with professional/managerial/executive occupations, median value of owner-occupied housing units, and percent of families above poverty line. This model does not include the NSES score.
[g] Additionally adjusted for the NSES score and all individual SES indicators.
[h] Additionally adjusted for all individual and neighborhood SES indicators except for NSES score.

Table S5. Estimated hazard ratios for time to first cardiovascular event associated with 5 μg/m^3 higher exposure to baseline $PM_{2.5}$ from multiple imputation analysis. [a,b]

	HR[a]	(95% CI)
$PM_{2.5}$ without SES measures	1.12	(1.01, 1.24)
Individual SES		
Education	1.12	(1.02, 1.24)
Income	1.12	(1.01, 1.23)
Occupation	1.12	(1.01, 1.24)
All Individual SES[c]	1.12	(1.02, 1.24)
NSES		
Education	1.12	(1.01, 1.24)
Income	1.12	(1.01, 1.23)
Employment	1.12	(1.01, 1.24)
Home values	1.12	(1.01, 1.23)
Poverty	1.12	(1.01, 1.24)
NSES score	1.12	(1.01, 1.24)
All NSES (no NSES score)[d]	1.13	(1.02, 1.25)
Individual SES and NSES		
All individual SES + NSES score[e]	1.13	(1.03, 1.25)
All individual SES + All NSES (no NSES score)[f]	1.13	(1.02, 1.24)

$PM_{2.5}$, fine particulate matter; HR, hazard ratio; CI, confidence interval; SES, socioeconomic status; NSES, neighborhood socioeconomic status.

[a] All HRs are adjusted for age, race/ethnicity, smoking, body mass index, diabetes, hypertension, and hypercholesterolemia.

[b] Models listed are separate from one another and are not sequentially adjusted. The models adjust for SES measures indicated and no other SES measures listed above or below it. Models adjusting for combinations of SES measures (i.e. All Individual SES) are notated and explained below.

[c] Additionally adjusted for the following individual-level SES indicators: education, total family income, and occupation.

[d] Additionally adjusted for the following neighborhood-level SES indicators: percent adults over 25 years with high school degree, median family income, percent of civilian population over 16 with professional/managerial/executive occupations, median value of owner-occupied housing units, and percent of families above poverty line. This model does not include the NSES score.

[e] Additionally adjusted for the NSES score and all individual SES indicators.

[f] Additionally adjusted for all individual and neighborhood SES indicators except for NSES score.

Table S6. Estimated hazard ratios for time to first cardiovascular event associated with 5 μg/m^3 higher exposure to $PM_{2.5}$ according to levels of SES and *p*-values for interactions, from multiple imputation analysis.

	HR[a]	(95% CI)	Overall *P*-value[b]
Individual SES			
Education			0.063
Not high school graduate	1.43	(1.07, 1.91)	
High school graduate	1.01	(0.88, 1.16)	
Some college or associate degree	1.16	(1.04, 1.30)	
Bachelor's degree or higher	1.18	(0.99, 1.40)	
Family Income			0.047
<$20,000	1.26	(1.09, 1.45)	
$20,000-$34,999	1.02	(0.90, 1.16)	
$35,000-$49,999	1.29	(1.06, 1.58)	
$50,000-$74,999	1.01	(0.85, 1.20)	
$75,000+	1.10	(0.92, 1.32)	
Occupation at baseline			0.853
Managerial/professional	1.18	(1.03, 1.36)	
Technical/sales/administrative	1.11	(0.96, 1.30)	
Service/labor	1.09	(0.92, 1.29)	
Homemaker only	1.11	(0.84, 1.46)	
Neighborhood SES			
NSES score[c]			0.002
<72.7	1.42	(1.26, 1.60)	
72.7-77.8	1.08	(0.95, 1.21)	
77.9-81.9	1.02	(0.86, 1.23)	
≥82.0	1.00	(0.81, 1.22)	
Percent adults 25+ with HS degree			0.188
<82.3%	1.00	(1.01, 1.35)	
82.3-89.4%	1.00	(1.08, 1.44)	
89.5-94.3%+	1.00	(0.91, 1.38)	
>94.3%	1.00	(0.86, 1.17)	
Median family income			0.068
<$47,891	1.29	(1.15, 1.45)	
$47,891-62,526	1.17	(1.05, 1.30)	
$62,527-81,973	1.07	(0.89, 1.28)	
>$81,973	0.97	(0.82, 1.14)	
Percent civilians over 16 with professional/managerial/executive occupations			0.409
<29.7%	1.26	(1.09, 1.45)	
29.7-41.3%	1.18	(1.06, 1.32)	
41.4-54.1%	0.97	(0.76, 1.23)	
>54.1%	1.12	(0.93, 1.35)	

Median home value			<0.0001
<$103,500	1.43	(1.26, 1.63)	
$103,500-153,599	1.22	(1.04, 1.43)	
$153,600-233,999	1.00	(0.89, 1.13)	
>$233,999	0.91	(0.77, 1.07)	
Percent families above poverty line			0.0001
<89.2%	1.36	(1.22, 1.52)	
89.2-94.0%	1.08	(0.92, 1.26)	
94.1-96.5%	1.03	(0.88, 1.21)	
>96.5%	1.06	(0.88, 1.28)	

$PM_{2.5}$, fine particulate matter; SES, socioeconomic status; HR, hazard ratio; CI, confidence interval; NSES, neighborhood socioeconomic status; HS, high school.

[a] All HRs (except those for the NSES score) are adjusted for age, race/ethnicity, smoking, body mass index, diabetes, hypertension, hypercholesterolemia, and all individual and neighborhood socioeconomic status indicators except for the NSES score.

[b] *P*-value for joint test of all multiplicative interaction terms.

[c] The NSES score model adjusted for individual level SES indicators but no other NSES indicators.

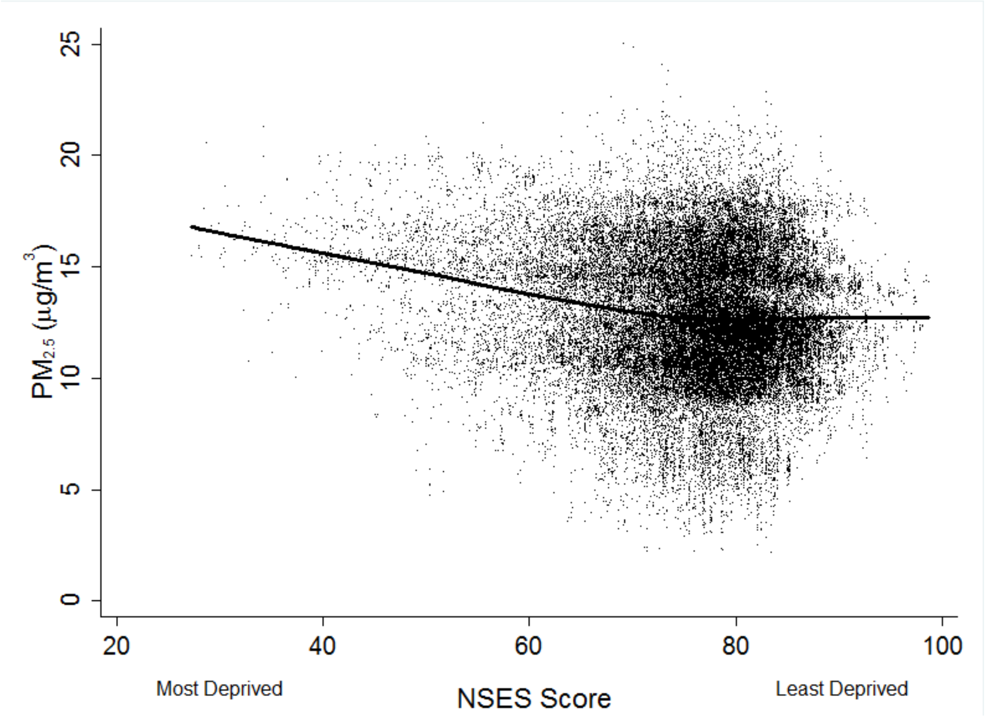

Figure S1. Scatter plot of first predicted annual average $PM_{2.5}$ by neighborhood socioeconomic status (NSES) score with a locally weighted scatterplot smoothing (LOWESS) curve. For most participants, the first available $PM_{2.5}$ prediction was the baseline prediction; otherwise, the next available non-missing $PM_{2.5}$ prediction was used.

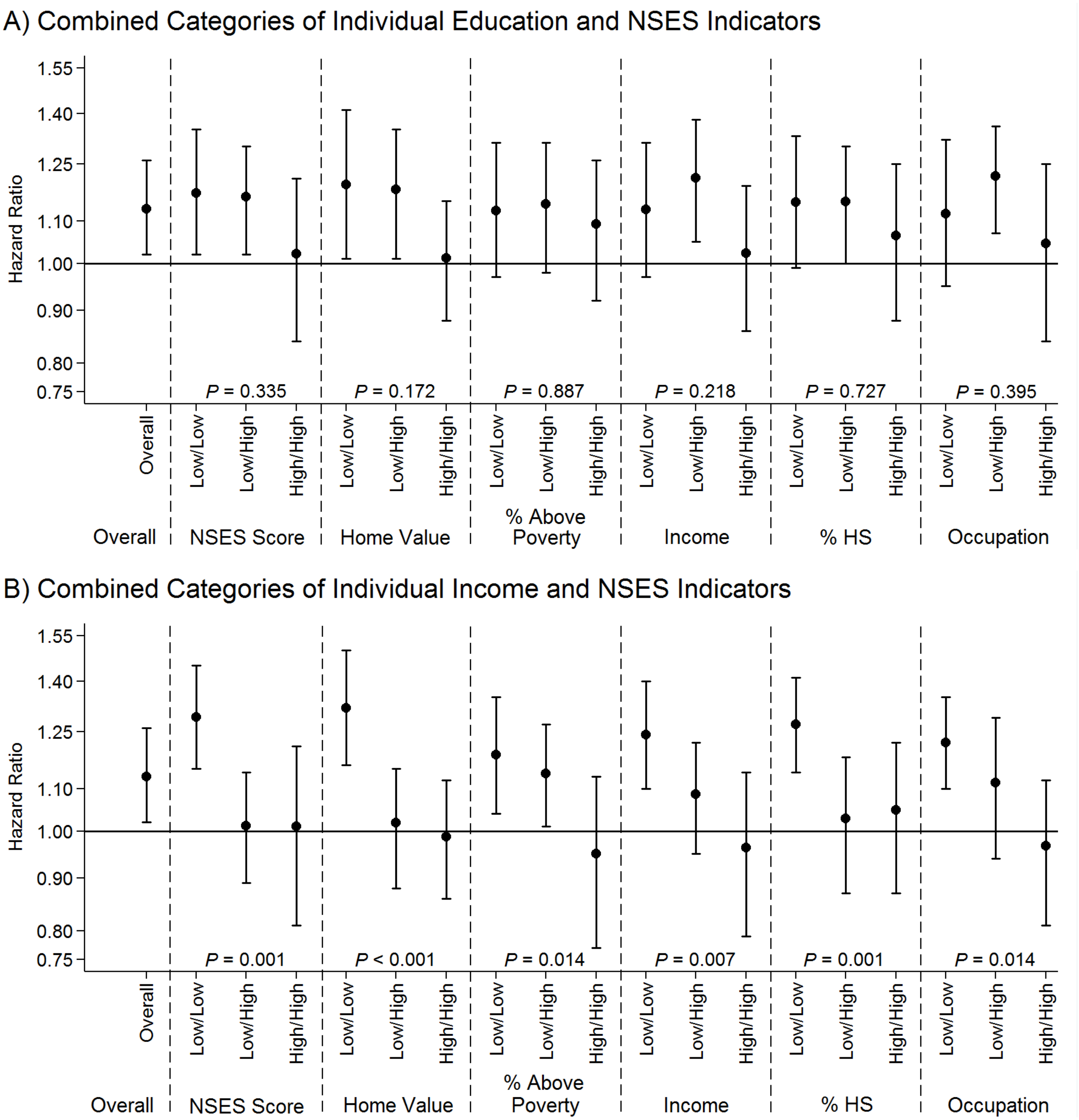

Figure S2. Estimated Hazard Ratios for Time to First Cardiovascular Event Associated with 5 μg/m^3 Higher Exposure to Fine Particulate Matter ($PM_{2.5}$) According to Combined Levels of Individual and Neighborhood Socioeconomic Status (SES) and *P* values for interactions, Women's Health Initiative Observational Study, 1993-2005. All models except for the NSES score model (see below) adjusted for age, race/ethnicity, smoking, body mass index, diabetes, hypertension, hypercholesterolemia, individual education, individual family income, individual occupation, percent adults over 25 years with high school degree, median family income, percent of civilian population over 16 with professional/managerial/executive occupations, median value of owner-occupied housing units, and percent of families above poverty line. Effect modification was modeled by adding multiplicative interaction terms. The NSES score model adjusted for individual SES indicators but not other NSES indicators. A) Estimated hazard ratios by levels of combined NSES and individual education. Low/Low

participants have low individual education and low neighborhood SES (NSES). Low/High participants have low education and high NSES or high education and low NSES. High/High participants have high education and high NSES. B) Estimated hazard ratios by levels of combined NSES and individual income. Low/Low participants have low individual income and low NSES. Low/High participants have low income and high NSES or high income and low NSES. High/High participants have high income and high NSES.
